# 229. IDSA FEATURED ORAL ABSTRACT: MVA-BN Induces a Low Avidity, Non-Durable Humoral Response Against Mpox Virus

**DOI:** 10.1093/ofid/ofae631.082

**Published:** 2025-01-29

**Authors:** Angelica C Kottkamp, Aaron Oom, Kesi K Wilson, Miilani E Yonatan, Stephanie Rettig, Heekoung Youn, Michael Tuen, Hayley Belli, Jane R Zucker, Jennifer Rosen, Marie I Samanovic, Ralf Duerr, Mark J Mulligan

**Affiliations:** NYU Grossman School of Medicine, New York, NY; New York University Grossman School of Medicine, New York, New York; NYU Grossman School of Medicine, New York, NY; NYU Langone Health, New York, New York; NYU Langone Vaccine Center, New York, New York; Nyusim, New York, New York; NYU Langone Health, New York, New York; New York University Grossman School of Medicine, New York, New York; NYC Department of Health and Mental Hygiene, Long Island City, New York; NYC Department of Health and Mental Hygiene, Long Island City, New York; NYU Grossman School of Medicine, New York, NY; NYU Langone Health, New York, New York; NYU Grossman School of Medicine, New York, NY

## Abstract

**Background:**

The 2022 global outbreak of clade IIb mpox, the first significant occurrence outside Africa, led to vaccination campaigns using the third-generation orthopoxvirus vaccine modified vaccinia Ankara, Bavarian Nordic (MVA-BN or JYENNEOS vaccine). In the US, a one-fifth dose of an intradermal regimen of the MVA-BN was authorized as a dose-sparing alternative to the two-dose subcutaneous regimen licensed in 2019. During the vaccination campaign, we launched the New York City Observational Study of Mpox Immunity (NYC OSMI) which enrolled participants who received the MVA-BN vaccine during the outbreak in NYC. Here we report the antibody data at one-year post vaccination.

**Figure:**
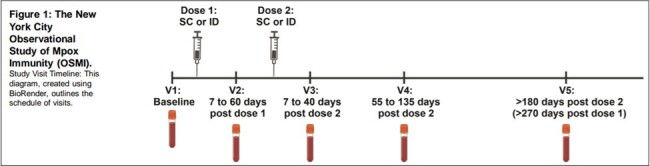

**Methods:**

We initiated the New York City Observational Study of Mpox Immunity (NYC OSMI), a longitudinal study involving 171 MVA-BN vaccinees, some with prior smallpox vaccination and mpox convalescents. Study participants had blood drawn prior to vaccination, after one dose, and after two doses. We performed Immunofluorescence-based Mpox virus (MPXV) clade IIb microneutralization assays, Multiplexed immunoassay for binding antibodies and avidity, and Anti-MPXV H3 Enzyme-Linked Immunosorbent Assay (ELISA). Paired measurements in figures were analyzed by Wilcoxon matched-pairs signed rank test. Comparisons of multiple sample groups in figures were conducted by Kruskal-Wallis test with Dunn’s method for multiple comparisons. Linear mixed-effects regressions (LMER) were used to model neutralizing titer durability with repeated measurements.

**Figure:**
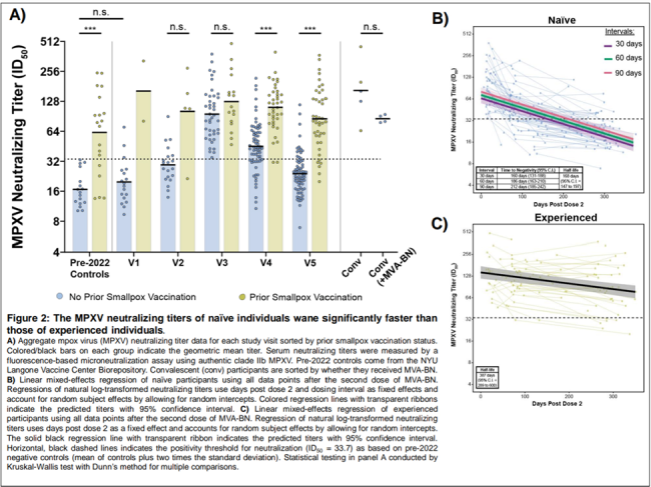

**Results:**

Our findings indicate that neutralizing titers in naïve individuals return to baseline in less than a year, while those with prior smallpox vaccination maintain elevated titers. Both groups generate robust IgG responses against MPXV H3 and A35, but the response in naïve vaccinees, characterized by lower avidity, is short-lived.

**Figure:**
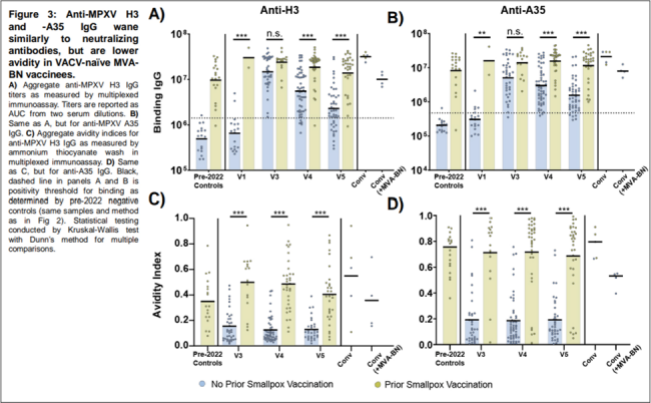

**Conclusion:**

These results highlight the necessity for further investigation into MVA-BN's long-term protection, potential need for booster doses, and the development of next-generation orthopoxvirus vaccines.

**Disclosures:**

**Mark J. Mulligan, M.D.**, HilleVax: Advisor/Consultant|Meissa Vaccines: Advisor/Consultant|Pfizer: Advisor/Consultant|Pfizer: Grant/Research Support|Sanofi: Grant/Research Support

